# Plasmonic Cavities and Individual Quantum Emitters in the Strong Coupling Limit

**DOI:** 10.1021/acs.accounts.2c00028

**Published:** 2022-06-01

**Authors:** Ora Bitton, Gilad Haran

**Affiliations:** † Chemical Research Support, Weizmann Institute of Science, P.O. Box 26, Rehovot 7610001, Israel; ‡ Department of Chemical and Biological Physics, Weizmann Institute of Science, P.O. Box 26, Rehovot 7610001, Israel

## Abstract

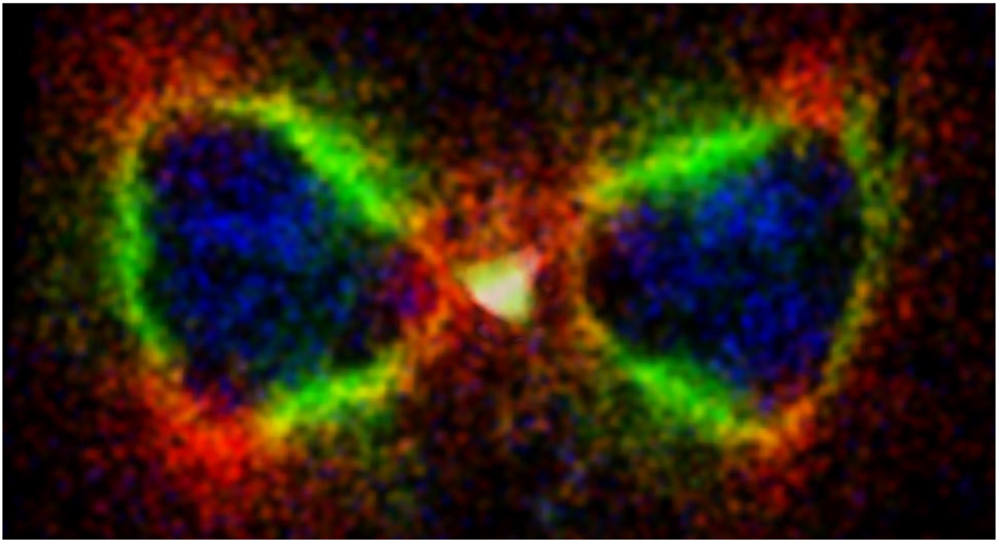

The interaction of emitters with plasmonic cavities (PCs) has been studied extensively during the past decade. Much of the experimental work has focused on the weak coupling regime, manifested most importantly by the celebrated Purcell effect, which involves a modulation of the spontaneous emission rate of the emitter due to interaction with the local electromagnetic density of states. Recently, there has been a growing interest in studying hybrid emitter-PC systems in the strong-coupling (SC) regime, in which the excited state of an emitter hybridizes with that of the PC to generate new states termed polaritons. This phenomenon is termed vacuum Rabi splitting (VRS) and is manifested in the spectrum through splitting into two bands.

In this Account, we discuss SC with PCs and focus particularly on work from our lab on the SC of quantum dots (QDs) and plasmonic silver bowtie cavities. As bowtie structures demonstrate strong electric field enhancement in their gaps, they facilitate approaching the SC regime and even reaching it with just one to a few emitters placed there. QDs are particularly advantageous for such studies, due to their significant brightness and long lifetime under illumination. VRS was observed in our lab by optical dark-field microspectroscopy even in the limit of individual QDs. We further used electron energy loss spectroscopy, a near-field spectroscopic technique, to facilitate measuring SC not only in bright modes but also in subradiant, dark plasmonic modes. Dark modes are expected to live longer than bright modes and therefore should be able to store electromagnetic energy for longer times.

Photoluminescence (PL) is another useful observable for probing the SC regime at the single-emitter limit, as shown by several laboratories. We recently used Hanbury Brown and Twiss interferometry to demonstrate the quantum nature of PL from QDs within PCs, verifying that the measurements are indeed from one to three QDs. Further spectroscopic studies of QD-PC systems in fact manifested several surprising features, indicating discrepancies between scattering and PL spectra. These observations pointed to the contribution of multiple excited states. Indeed, using model simulations based on an extended Jaynes–Cummings Hamiltonian, it was found that the involvement of a dark state of the QDs can explain the experimental findings. Given that bright and dark states couple to the cavity with different degrees of coupling strength, the PC affects in a different manner each excitonic state. This yields complex relaxation pathways and interesting dynamics.

Future work should allow us to increase the QD-PC coupling deeper into the SC regime. This will pave the way to exciting applications including the generation of single-photon sources and studies of cavity-induced coherent interactions between emitters.

## Key References

- Santhosh, K.; Bitton, O.; Chuntonov, L.; Haran, G. Vacuum Rabi splitting in a plasmonic cavity at the single quantum emitter limit. *Nat. Commun*. **2016**, *7*, 11823.^1^ Vacuum Rabi splitting, a manifestation of strong coupling, is observed in the scattering spectra of silver bowtie plasmonic cavities loaded with one to a few quantum dots. The observations are verified by polarization-dependent experiments and electromagnetic calculations.
- Bitton, O.; Gupta, S. N.; Houben, L.; Kvapil, M.; Křápek, V.; Šikola, T.; Haran, G. Vacuum Rabi splitting of a dark plasmonic cavity mode revealed by fast electrons. *Nat. Commun*. **2020**, *11*, 487.^2^ Electron energy loss spectra can also reveal strong coupling between quantum dots and a plasmonic bowtie. Surprisingly, a dark mode of the cavities is found to strongly interact with the quantum emitters.
- Gupta, S. N.; Bitton, O.; Neuman, T.; Esteban, R.; Chuntonov, L.; Aizpurua, J.; Haran, G. Complex plasmon-exciton dynamics revealed through quantum dot light emission in a nanocavity. *Nat. Commun*. **2021**, *12*, 1310.^3^ The excited-state complexity of quantum dots is revealed by their interaction with plasmonic cavities and photoluminescence spectra. A dark state of the quantum dots is enhanced by the cavity and appears in the spectra, as revealed by a careful theoretical analysis.
- Bitton, O.; Gupta, S. N.; Cao, Y.; Haran, G. Improving the quality factors of plasmonic silver cavities for strong coupling with quantum emitters. *J. Chem. Phys.***2021**, *154*, 014703.^4^ Strong coupling in plasmonic devices is limited by losses in the metal. A reduction in the thickness of the chromium adhesion layer used to prepare plasmonic bowties can decrease the line widths of both bright and dark plasmonic modes by a large factor, up to 2. We show that this facilitates reaching the strong-coupling regime with quantum dots.

## 1 Introduction

Interactions between resonant optical cavities and quantum emitters (QEs) have attracted much interest over the past decades.^5−8^ Specifically, light–matter interactions on the nanoscale have become a subject of rapidly increasing scientific importance, paving the way for the control and manipulation of both light and matter well below the diffraction limit. Studies of absorption and emission of light by atoms and molecules within cavities form a platform for enhancing our understanding of the chemical properties of matter. They also allow us to alter fundamental material properties via the formation of hybrid light–matter polaritonic states, leading to the emergence of the new field of polaritonic chemistry,^9^ with a high potential for multiple exciting applications.^10^

**Figure 1:**
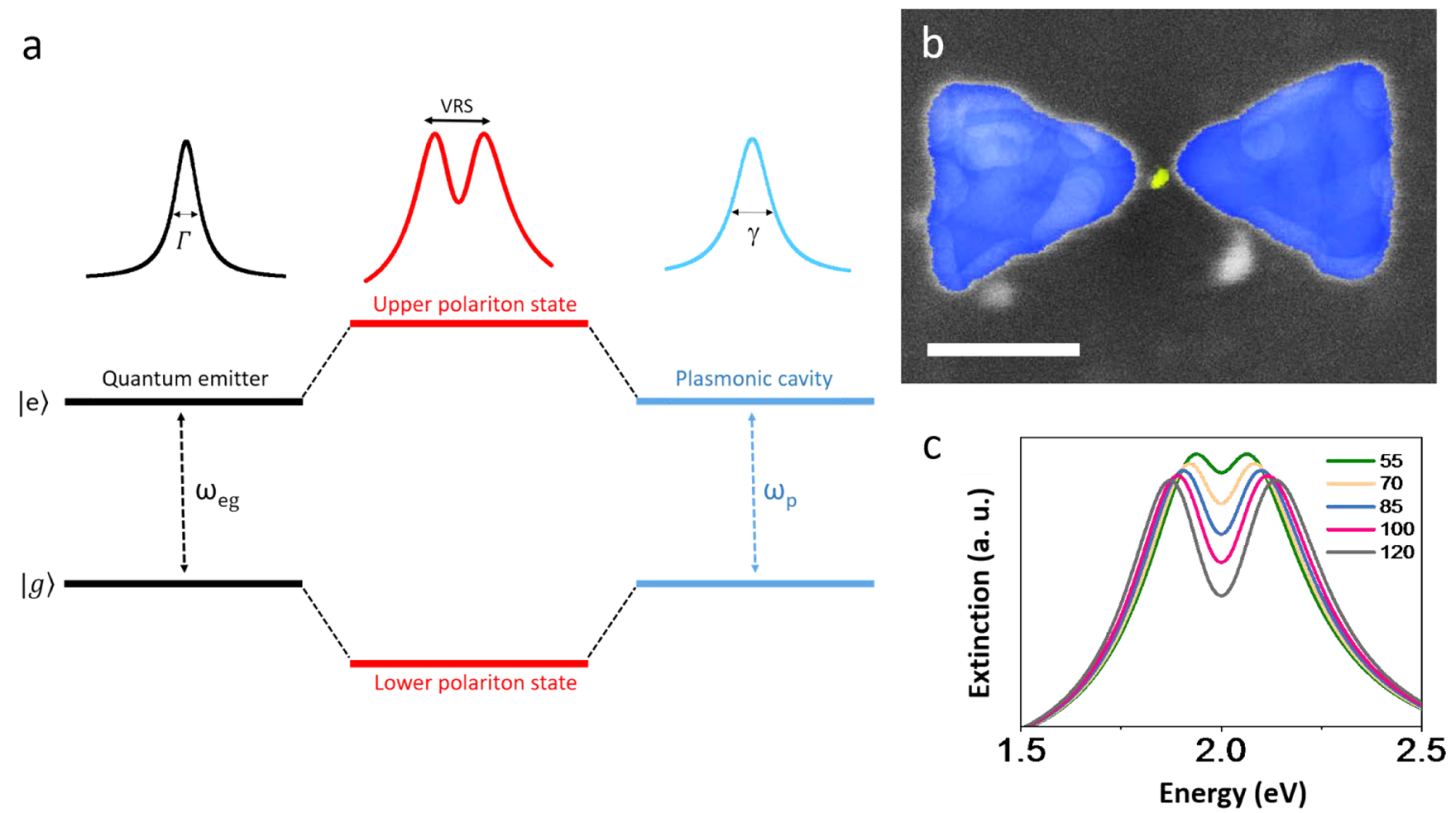
The strong coupling regime. (a) Schematic of resonance interaction between a two-level quantum emitter and a confined electromagnetic field in a cavity, which results in two new hybrid polaritonic states, separated by twice the coupling. This leads to the appearance of vacuum Rabi splitting (VRS) in experimentally observable spectra. (b) False-color scanning electron micrograph of a silver bowtie cavity coupled to a single quantum dot. Scale bar represents 50 nm. (c) Coupled-oscillator model simulations of extinction spectra with increasing coupling strengths, with values given in the legend in meV. Reproduced with permission from ref (2). Copyright 2020, the authors. Published by Springer Nature under a Creative Commons CC BY License.

A QE positioned within a cavity can be either weakly or strongly coupled to it, depending on the size of the coupling strength, *g*, compared to the damping rate of the emitter, Γ, and that of the cavity, γ. When the cavity and emitter are weakly coupled, the eigenstates of the two parts of the system are not modified. However, spontaneous emission within the cavity may be enhanced due to the increased local density of states (LDOS), compared to free space; this is the well-known Purcell effect.^11^ When the cavity and emitter are strongly coupled, their wave functions mix, and the eigenstates of the coupled system are different than those of the emitter and the cavity individually. Here, the coupling modifies the energy spectrum of the hybrid system. The excited states of the emitter and cavity form two new modes with new frequencies, called polaritons, Ω_+_ and Ω_–_, whose properties can be derived by various theoretical approaches,^6^ such as the coupled oscillator model.^12^ The energy difference between the upper and lower polariton is called vacuum Rabi splitting (VRS)^13^ and is manifested as a dip in the system’s spectral response. A schematic illustration of resonance interaction between a two-level QE and a confined electromagnetic field in a cavity is shown in Figure 1a.

The coupling strength, *g*, is related to the rate of energy transfer between the cavity and emitter and is defined as the scalar product of the dipole moment of the QE’s exciton μ_ex_ and the electric field *E* at its position *g* = μ_ex_ × *E*. An increase of *g* can then be achieved either by using excitons with a larger dipole moment or by increasing the electric field strength at the position of the emitter. One way to parametrize the latter is in terms of the “Purcell factor”, defined as the ratio of the quality factor of the cavity, *Q*, to the mode volume *V*, *Q*/*V*. *Q* in turn is the ratio of the cavity’s resonance frequency to its spectral width.

The strong coupling (SC) regime has been of much interest in physics in recent decades, as it has led to the discovery of various breakthrough phenomena, including polariton lasing^14^ and photon blockades,^15^ as well as Bose–Einstein condensation^16^ and superfluidity of polaritons.^17^ Also, SC has the potential to be of importance for a broad range of applications in quantum information processing,^18^ quantum computing,^19^ and single photon sources.^20^ Strong light–matter interactions have also been proposed to facilitate modulation of relaxation pathways as well as rates of chemical reactions and charge and energy transfer.^9,10,21^

Dielectric cavities, whose *Q* values can be very high (often many thousands), have been traditionally used in studies of SC.^13,22^ The size of a dielectric cavity cannot be smaller than half the wavelength. This limits how small the mode volume can be in this type of cavity and therefore mandates a very high *Q* in order to reach the SC regime. The smallest unitless effective mode volume (i.e., the ratio of mode volume to resonant wavelength cubed, *V*_eff_ = *V*/λ^3^) for these cavities is *V*_eff_ ∼ 1 (ref (23)).

More recently, plasmonic cavities (PCs) have been introduced, and while they are characterized by small *Q* values due to high losses in metal (*Q* ∼ 10), their effective mode volumes can be as small as *V*_eff_ ∼ 10^–6^ (ref (22)). This enables reaching the SC regime even at room temperature. An excellent structure to serve as a PC is the gap between metallic NPs that are brought in close proximity. This gap, commonly termed a “hotspot”, sustains a very large field enhancement.^24,25^ An example is a dimer composed of two spheres or a bowtie structure.^22^ Interestingly, a PC can also be based on a nanoparticle-on-mirror configuration, i.e., a single particle such as a nanocube^26^ or sphere^27^ that is coupled to its mirror image by positioning it close to a flat metallic surface. Various types of QEs have been used for SC studies with PCs, including dye molecules, J-aggregates of cyanine dyes, nitrogen vacancy (NV) centers in diamonds, and QEs in 2D materials.^22^

In this Account, we focus on one type of QEs, semiconductor nanocrystals, or quantum dots (QDs).^28^ The spectroscopic features of QDs involve transitions between discrete, 3D particle-in-a-box states of both electrons and holes. QDs offer a variety of advantages for SC studies. First, they have relatively large transition dipole moments, corresponding to strong oscillator strengths. The typical transition dipole moment of fluorescent molecules is ∼1D (Debye), while for QDs it can be 10 times larger.^29^ Further, while early types of QDs suffered from low emission quantum yields and blinking,^30^ core–shell QDs (e.g., CdSe/ZnS, as used in our studies) have been engineered to confine excitons within the core, making them less sensitive to quenching reactions involving surface states^31^ and therefore rendering them brighter and more stable.

Much work has been devoted to studying the interaction of QDs with PCs within the weak coupling regime,^8^ and only in recent years has research on the SC regime emerged^8,1,32−35^ (see Figure 6 in ref (8) for an illustration that summarizes these achievements by positioning them on a continuous scale of coupling strength, *g*).

We discuss below our research on SC between one and a few QDs and silver plasmonic bowtie PCs (Figure 1b). QD–bowtie compound systems have been studied in our lab using several spectroscopic techniques, including single-particle light scattering, electron energy loss spectroscopy (EELS), and photoluminescence (PL) spectroscopy. In general, we find that for both bright and dark plasmonic modes, the SC regime can be achieved with a few QDs in the cavity, and even at the level of the single QD these devices are situated at the onset of the SC regime.

## 2 Observing Vacuum Rabi Splitting

### Criteria for Strong Coupling

As discussed in detail, e.g., in ref (6), in order to have two real polaritonic states of a coupled cavity–QE system (i.e., two real solutions for the coupled Hamiltonian), the following criterion should be met:
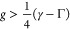
1When this criterion is fulfilled, the system has passed an exceptional point^36^ and is therefore guaranteed to possess two distinct eigenstates. However, the criterion does not guarantee two well-separated peaks in the spectrum, due to finite line widths of the plasmon and emitter peaks, related to their damping rates, which can conceal the dip due to VRS. A more strict criterion considers SC to occur only when at least one complete Rabi oscillation occurs:^6,13^
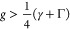
2

This criterion ensures that the measured spectra will clearly show these states as separate peaks. In reality, there is no sharp threshold above which SC occurs. Rather, there is a smooth and continuous evolution of a spectral shape in which the polaritons gradually develop to form two separate solutions (Figure 1c).

### Scattering Spectroscopy

**Figure 2:**
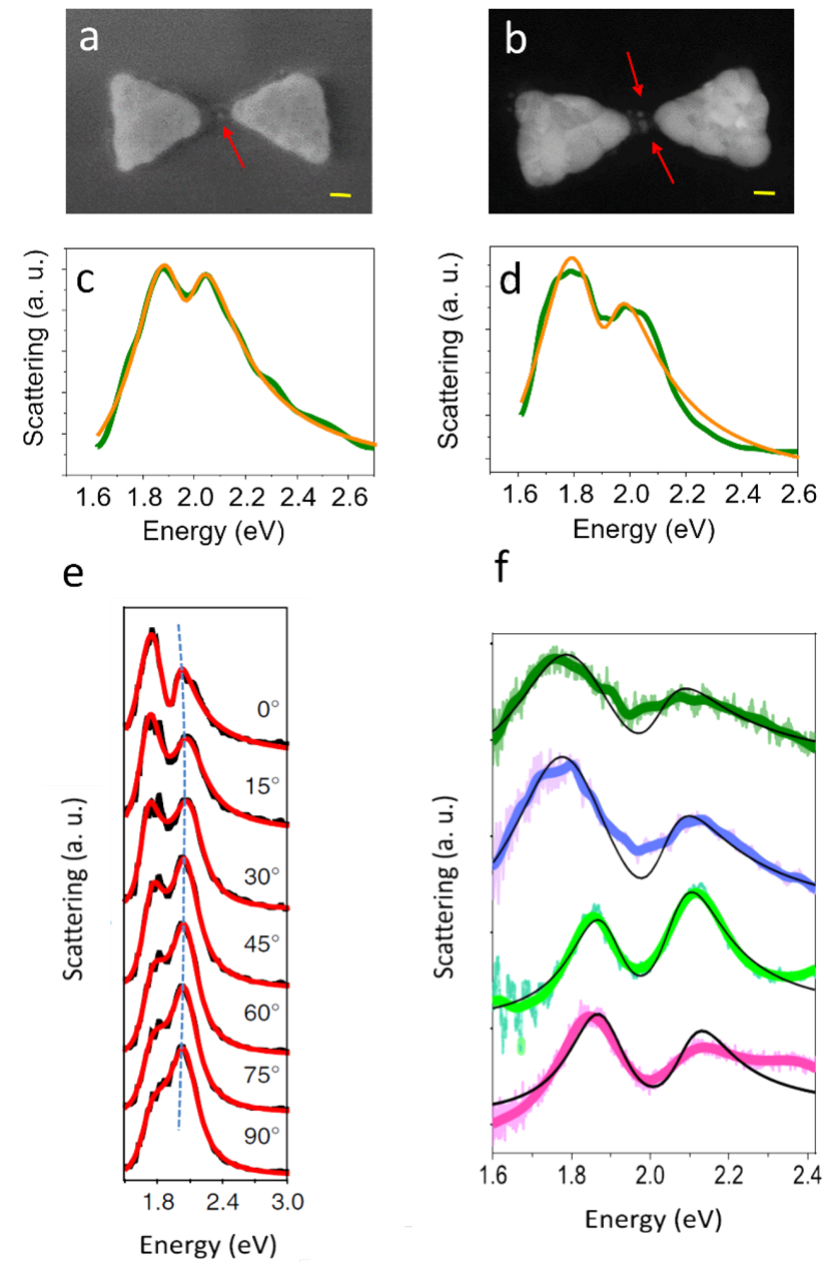
Scattering spectra of bowties coupled to QDs. (a–d) Electron micrographs (panels a,b) and dark-field scattering spectra (panels c,d) of two bowties containing single and double QDs. Scale bars represent 10 nm. The orange lines in panels c and d are fits to the coupled-oscillator model. Reproduced with permission from ref (3). Copyright 2021, the authors. Published by Springer Nature under a Creative Commons CC BY License. (e) Polarization-dependent scattering measurements of a bowtie coupled to QDs. Reproduced with permission from ref (1). Copyright 2021, the authors. Published by Springer Nature under a Creative Commons CC BY License. (f) Scattering spectra from bowtie PCs with QDs in their gap prepared with a 3 nm Cr layer (dark green and purple) and without Cr (light green and pink). Transparent and solid color curves are raw and smoothed data, respectively, and black lines are fits to the coupled-oscillator model. Reproduced with permission from ref (4). Copyright 2021 AIP Publishing LLC.

To observe SC between bowtie structures and a small number of QDs (down to one, Figure 1b), we developed a process by which QDs could be inserted into bowtie gaps. Using interfacial capillary forces, QDs were pushed into holes generated in a resist layer right at the center of the bowties.^1^ Examples of two such devices are shown in Figure 2a,b. Coupled QD-bowtie devices were measured using dark-field microspectroscopy. Scattering spectra measured with nonpolarized light for bowties coupled to one and two QDs are shown in Figure 2c and d, respectively. The spectra show a splitting, as one would expect in the SC regime. Spectra were fitted to an expression based on the coupled-oscillator model.^12^ (See Methods section in ref (1) for the explicit expression we used.) Since the values of γ and Γ were independently measured to be 395 and 130 meV, respectively, we calculated that the first criterion for SC (eq 1) is fulfilled when *g* > 66 meV and the second criterion (eq 2) when *g* > 131 meV. The extracted values of *g* obtained from the fits reveal that the sample presented in Figure 2d (with *g* = 103 meV) fulfills the first criterion for SC while the sample presented in Figure 2c (with *g* = 55 meV) is only close to fulfilling this. The value of *g* obtained in our experiment can be as high as 200 meV, but most values are within the range of 50–110 meV, mostly fulfilling the first criterion of SC, but not the second.

Polarization-dependent measurements showed that when the polarization of light is gradually rotated from the direction parallel to the bowtie axis to the orthogonal direction, the dip in the spectrum disappears and only a single peak, corresponding to the spectrum of a single prism, is left (Figure 2e).^1^ This proves that the obtained splitting is due to coupling between the QDs and the longitudinal mode of the plasmon.

As discussed in section 1, a high coupling rate is not the only key parameter that determines entering the SC regime. With a fixed coupling rate, SC can also be approached by reducing the plasmon and QD damping rates. We recently found that the thickness of the adhesion layer under the metal bowties dramatically affects the plasmon damping rate (plasmon line width) and hence allows us to fulfill more easily the criteria for SC (eq 1 and eq 2).^4^ We therefore integrated QDs into silver bowtie PCs fabricated either with or without an adhesion layer. The two upper spectra in Figure 2f (dark green and purple curves) were measured for devices with a 3 nm Cr adhesion layer, while the two lower spectra (light green and pink curves) were measured for devices without an adhesion layer. Fits to the coupled-oscillator model showed that the removal of the adhesion layer reduced the plasmon line width by >50%, making the SC-related dip in the spectrum more prominent, as expected based on the second criterion for SC, eq 2.

The observation of a dip in a scattering spectrum requires some caution before it can be attributed to VRS. It is possible, for example, that this dip is not due to SC but rather due to enhanced absorption, a well-known effect in the weak coupling regime.^37^ This possibility is more relevant to experiments in which a large number of QEs are inserted into a PC and less important in the case of the single-emitter limit. Also, splitting in scattering can be due to Fano-like interference between the contributions of the plasmon and QD dipoles. In principle, fitting to a model, such as the coupled-oscillator model, discriminates between interference and genuine SC. An experimental way to rule out the possibility of a contribution from interference would be to measure absorption or extinction spectra and observe VRS dips in these as well.^38,39^ However, it might be quite difficult to measure absorption from individual PC-QD devices. EELS spectroscopy can serve as a proxy for optical extinction spectroscopy,^40^ as will be discussed in the next subsection. As opposed to scattering, PL is an incoherent process and does not suffer from interferences. Therefore, splitting in the PL spectrum occurs only in the SC regime and has therefore been recognized as a definitive signature of Rabi splitting. Studies of PL from individual QDs coupled to PCs in our lab and others’ will be discussed below.

### EELS

This advanced spectroscopic modality is an excellent tool to measure and characterize plasmonic excitations.^41^ It offers the unique combination of spatial and energy resolution over a broad spectral range. Being a near-field technique, EELS relaxes the common far-field selection rules and can reveal subradiant dark modes. The full-mode EEL spectrum of our PCs is demonstrated in ref (2).

It is intuitively easy to understand that the EEL signal is closely related to the optical extinction spectrum of the nanoparticle.^40^ Therefore, VRS obtained in EELS is a definitive signature for SC. The first application of EELS to detect SC was reported by Lu et al.^42^ in their study of ZnO excitons coupled to silver nanoparticles. In this work, the lower and upper polaritons were characterized, and a Rabi splitting of 170 meV was extracted from the data. Yankovich et al.^43^ characterized polaritons generated by a hybrid system composed of an individual silver nanoparticle and a few-layer WS_2_ flake.

**Figure 3:**
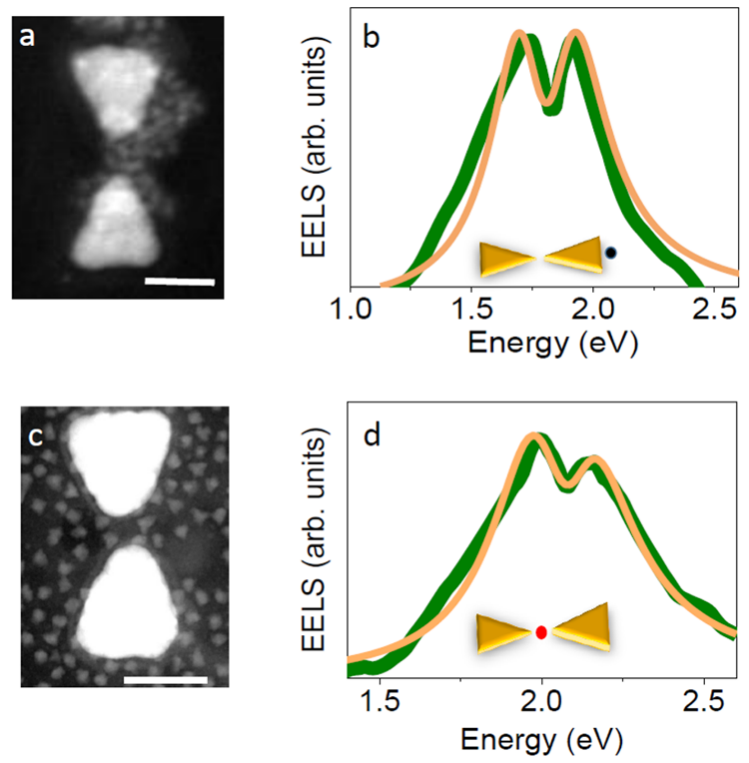
Coupling with bright and dark dipolar modes. (a, c) Electron micrographs of two devices loaded with QDs with an exciton energy of 1.8 and 2 eV, respectively. The scale bars represent 50 nm. (b, d) EEL spectra of the bright and dark dipolar modes of the devices in a and c, demonstrating Rabi splitting. Green curves are experimental data, and orange curves are fits to the coupled-oscillator model. Insets demonstrate the points of excitation by the electron beam. Reproduced with permission from ref (2). Copyright 2020, the authors. Published by Springer Nature under a Creative Commons CC BY License.

In our EELS studies of QD-PC coupling, we focused first on the lowest-energy dipolar bright mode of the PCs.^2^ We integrated into bowties QDs with an exciton energy of 1.8 eV, i.e., in resonance with that mode (Figure 3a). Locating the electron beam in the middle of the outer edge of the PCs, we measured spectra that demonstrated a Rabi splitting of ∼200 meV (Figure 3b). Fitting to a coupled-oscillator based function describing frequency-dependent extinction (yellow curve) revealed a coupling strength of *g* = 105 ± 2 meV.

We then focused on the lowest energy dipolar *dark* mode of the PCs by inserting into their gaps QDs possessing an emission energy of 2.0 eV (Figure 3c). Spectra measured with the electron beam located within the gap between the two parts of a PC, exciting the dark mode, demonstrated again Rabi splitting, with a splitting values of ∼160 meV and a *g* value as large as 83 ± 2 meV (Figure 3d). Therefore, even a dark mode that does not radiate to the far field can be strongly coupled to excitations of QEs, resulting in dark polaritonic states that can be probed only in the near field.

As noted above, in order to guarantee SC, one needs to position the QE where the electric field is highest within the cavity. Intriguingly, while for the bright mode the electric field was found to be concentrated within the gap, in between the prisms, and with the highest value close to the prism edge, for the dark mode the electric field was concentrated at the periphery of the gap. Indeed, we demonstrated that positioning a QE very close to the prism edge yields a larger coupling rate when coupled to the bright mode,^1^ and only if QDs were positioned at the periphery of the gap did splitting in the dark mode start to develop in the spectra.^2^

## 3 From Photoluminescence in a Cavity to Complex Photophysics

### Observing VRS with PL

**Figure 4:**
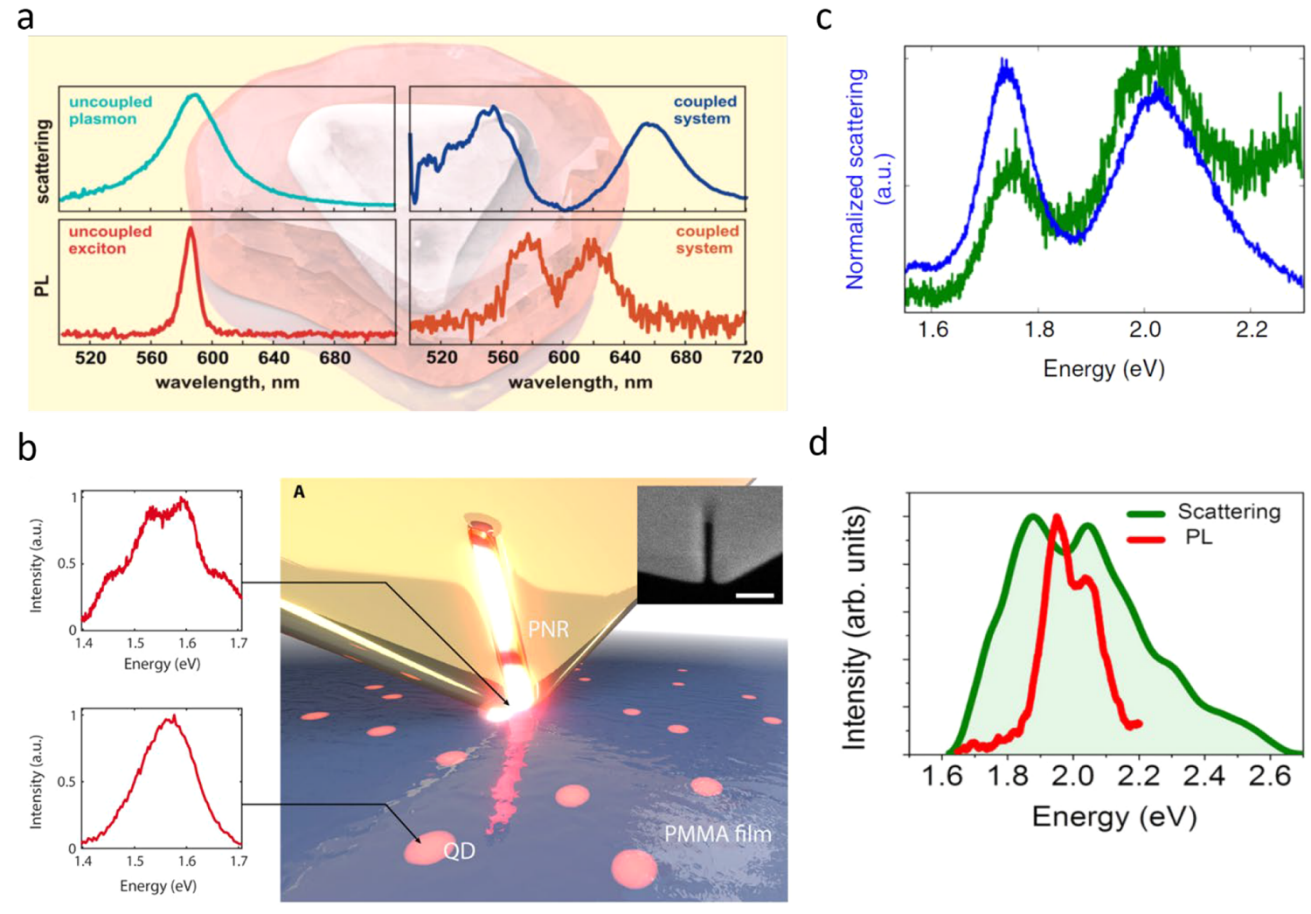
VRS demonstrated in photoluminescence. (a) VRS of up to 400 meV in scattering and photoluminescence spectra of an individual silver prism coupled to molecular J-aggregates. Reproduced with permission from ref (39). Copyright 2016 American Chemical Society. (b) Illustration of a plasmonic resonator at the tip of a scanning probe interacting with QDs embedded in a polymer film. Left panels: The spectrum of a QD changes significantly (top) when coupled to the resonator. Inset: Electron micrograph of the nanoresonator. Scale bar represents 100 nm. Reproduced with permission from ref (35). Copyright 2018, the authors, some rights reserved; exclusive licensee American Association for the Advancement of Science. No claim to original U.S. Government Works. Distributed under a Creative Commons Attribution License 4.0 (CC BY). (c) Scattering (blue) and PL spectra (green) of a plasmon-emitter system showing Rabi splitting. Reproduced with permission from ref (34). Copyright 2018, the authors. Published by Springer Nature under a Creative Commons CC BY License. (d) Dark-field scattering spectra (green) and PL spectra (red) of a coupled silver bowtie-QD system. Reproduced with permission from ref (3). Copyright 2021, the authors. Published by Springer Nature under a Creative Commons CC BY License.

As noted above, VRS should in principle also be observed in PL spectra. Indeed, PL as a true indication for SC has been demonstrated in several studies with multiple emitters,^39,44^ as well as at the single emitter level.^34,35,45,46^ Melnikau et al. revealed signatures of SC in the PL of J-aggregates coupled to plasmonic nanoparticles,^44^ and later, Wersäll et al. demonstrated SC even in J-aggregate PL spectra measured from individual nanoparticles (Figure 4a).^39^ Gross et al. showed PL splitting for a single QD coupled to a plasmonic nanoresonator constructed at the end of a tip.^34^ The PL spectrum showed an unexpected four-peak structure, which was attributed to emission from the charged and neutral excited states of the QD, both coupled strongly with the cavity (Figure 4b).^34^ Leng et al. observed VRS in both scattering and PL spectra measured from individual coupled QD-gap plasmon system (Figure 4c).^35^

To shed more light on the relation of scattering and PL spectra in (or close to) the SC regime, we measured spectra from more than 20 bowtie cavities loaded with either one or a few QDs.^3^ Scattering spectra were measured, as discussed above, with dark-field microspectroscopy, and PL spectra were measured following excitation with a CW laser at 532 nm. An example of scattering and PL spectra measured from the same device is shown in Figure 4d. In this particular device the splitting obtained in the scattering spectrum is 200 meV, with a *g* value of 52.6 ± 0.3 obtained through a fit to the coupled-oscillator model. The PL spectrum measured from the same device is significantly narrower than the scattering spectrum. Also, the splitting between peaks obtained from the PL spectrum is only 100 meV, much smaller than the splitting obtained in scattering. These discrepancies between scattering and PL spectra have been observed in all measured PCs.^3^

### The Quantum Nature of PL Emission in a Plasmonic Cavity

**Figure 5:**
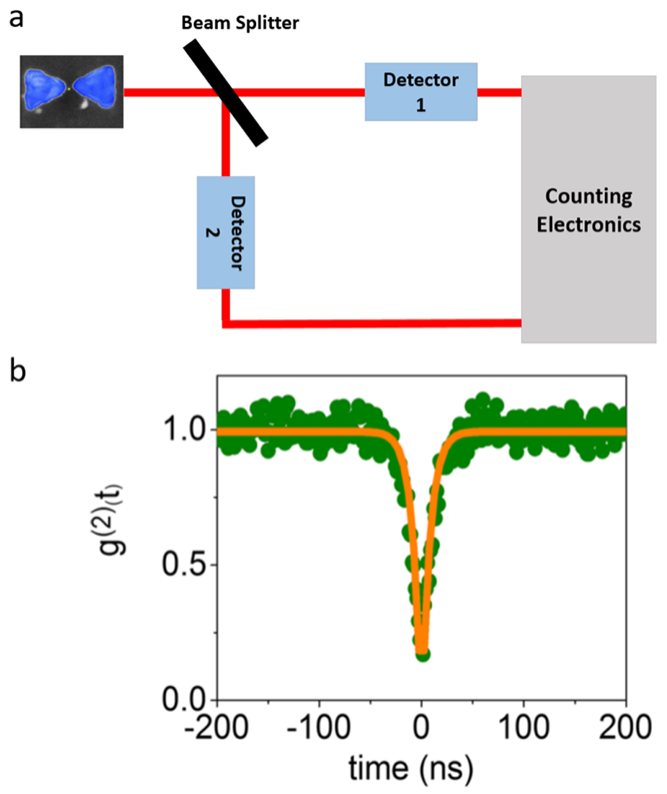
Second-order photon correlation functions of QDs coupled to a PC. (a) Schematic of the Hanbury Brown and Twiss experiment. (b) *g*^2^(*t*) of a single QD coupled to a bowtie manifesting antibunching. Reproduced with permission from ref (3). Copyright 2021, the authors. Published by Springer Nature under a Creative Commons CC BY License.

To shed further light on the phenomenology of PL in our PCs, we studied the nature of the emission through measurement of second-order correlation function *g*^2^(*t*) using Hanbury Brown and Twiss (HBT) interferometry. In this experiment (Figure 5a), the emitted light is split onto two channels and one observes the coincidence between the counts on two detectors. One asks, what is the probability of obtaining a photon on detector 2 at a time delay *t* following the detection of a photon on detector 1? For a single emitter, at a zero time delay, this probability vanishes, pointing to the sub-Poissonian nature of the emission and demonstrating its quantum origin.^47^

The time dependence of the second-order correlation curve is given by eq 3:
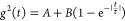
3Here, *A* and *B* are constants and τ is the excited-state lifetime of the QE. The signal at time zero is given approximately by 1 – 1/*N*, with *N* being the number of emitters in the cavity.^48^ By fitting a measured correlation curve to eq 3, both τ and the number of emitters *N* can be extracted. Indeed, the largest integer smaller than 1/(1 – *A*) provides an estimate for *N*.

We measured *g*^2^(*t*) of QDs trapped within PCs, and an example is shown in Figure 5b. The value of this correlation function at time zero suggests that the signal comes from a single QD. A fit to eq 3 provided a lifetime of 5 ns. *g*^2^(*t*) functions were measured from 16 of the devices whose scattering and PL spectra showed a clear indication of peak splitting, and the obtained lifetime values ranged between 3 and 12 ns, showing only a minor shortening compared to the lifetime of QDs on glass (∼24 ns).^3^ This finding was highly unexpected, as the mixing of the QD exciton with the plasmon in the cavity should have opened a fast relaxation channel with a lifetime closer to that of the plasmon.^49^

### Between Weak and Strong Coupling

**Figure 6:**
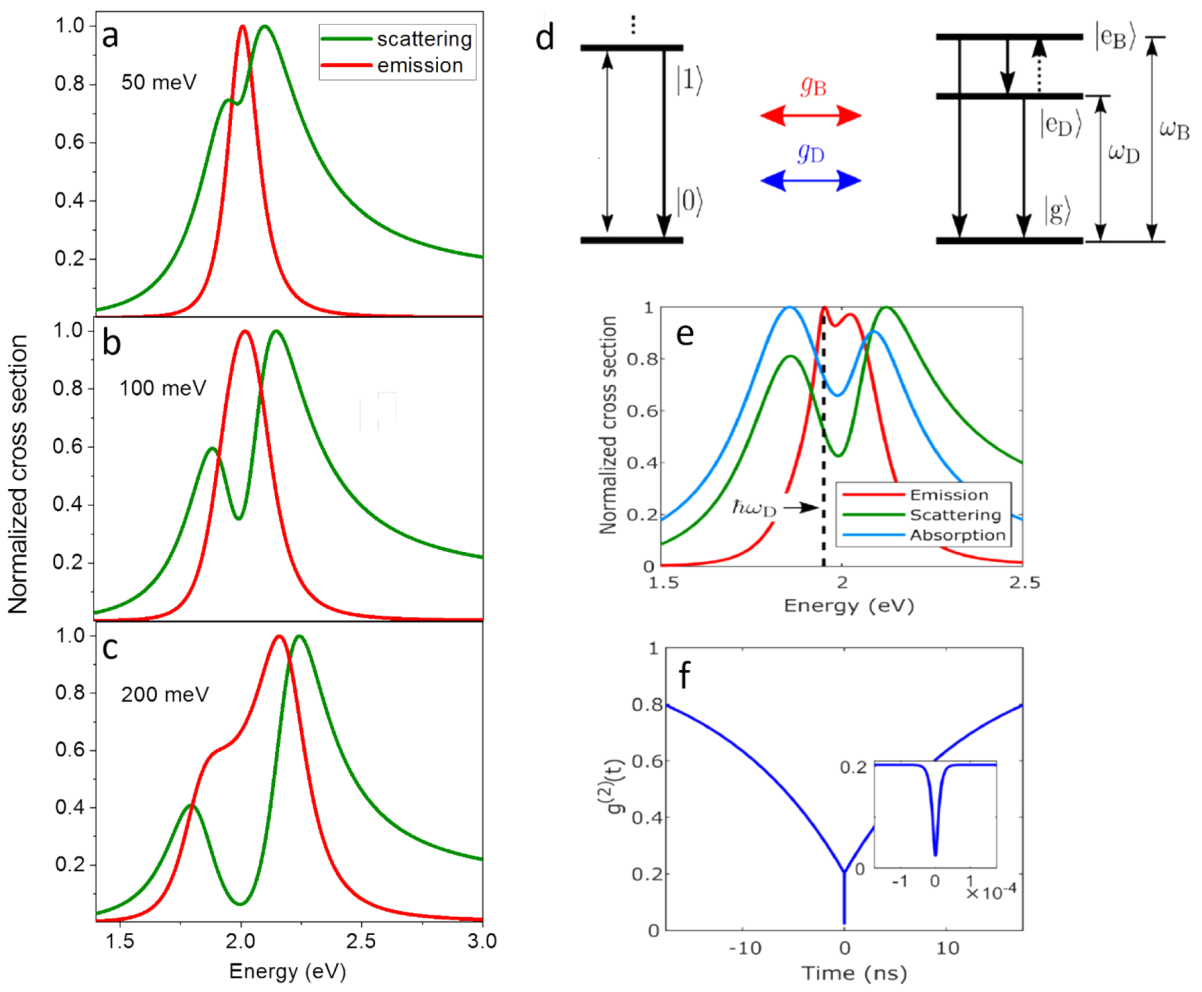
Complex plasmon-exciton dynamics in a bowtie-QD system. (a–c) Simulated scattering (green) and emission (red) spectra of a plasmonic cavity coupled to a QD described by a two-level bright electronic system. Here, the intrinsic decay rate of the plasmon is 400 meV, and that of the bright exciton is 0.1 μeV. The pure dephasing of the bright exciton is 130 meV. The exciton–plasmon coulpling is 50, 100, and 200 meV in a, b, and c, respectively. (d) Schematic level diagram describing the extended Jaynes–Cummings model. The plasmonic cavity is depicted on the left and the QD on the right. The QD is described as a three-level electronic system containing a ground state, |g⟩, a bright excitonic level, |e_B_⟩, and a dark excitonic level, |e_D_⟩. The bright (dark) excitonic transition occurs at energy ω_B_ (ω_D_). Plasmon–exciton coupling is described with rates *g*_B_ and *g*_D_ for coupling of the bright and dark exciton, respectively. (e) Emission (red), scattering (green), and absorption (blue) spectra calculated numerically with parameters shown in Table 1 in ref (3). The dashed line marks the energy of the dark exciton, ℏω_D_. (f) Simulated *g*^2^(*t*) features a two-component decay. Inset shows a zoom of the fast (femtosecond) decay of system excitations that is not resolved on the nanosecond time scale. (d–f) Reproduced with permission from ref (3). Copyright 2021 Springer Nature under a Creative Commons CC BY License.

In order to understand the discrepancies we found between scattering and PL measurements with regards to their broadening and splitting magnitude, it is instructive to understand how both PL and scattering develop in the strong coupling limit. For that, we simulated scattering and PL spectra using the Jaynes–Cummings model (Figure 6a–c). In Figure 6a, the SC regime is not yet attained. In Figure 6b, only the first criterion for SC (eq 1) is fulfilled. Here, the splitting in the scattering spectrum becomes evident, while the PL spectrum broadens with no signature of two peaks yet. When the second criterion for SC (eq 2) is also fulfilled (Figure 6c), two peaks appear in the PL spectrum as well, although it is still narrower than the scattering spectrum. Similar observations were presented by Leng et al., who explored the scattering and PL spectra in the weak, intermediate, and SC regime.^35^

As mentioned earlier, the *g* values obtained in our experiments mostly fulfill the first SC criterion, but not the second. The fact that PL spectra are much narrower than scattering spectra agrees with the results of the simulations presented in Figure 6b. However, only a single peak is expected in PL spectra in this regime, yet we observe two peaks in all measured spectra. Therefore, we considered a physical model that might lead to the appearance of two maxima in the PL spectra and might also explain the slow decay of PL in *g*^2^(*t*) curves. In particular, we recalled that QDs might possess dark, long-lived excited states originating from charge carriers trapped on the surface.^50^ The scheme of the plasmon and QE energy levels used in the model is shown in Figure 6d. The QE involves two excited states, i.e., the band-edge bright exciton of QDs and a dark state that sits below it by 50 meV. The bright and dark states can both couple to the cavity. However, the former is strongly coupled, while the latter is only weakly coupled. Using an extended Jaynes–Cummings Hamiltonian, we found that the coupling of the dark state to the cavity leads to the emergence of an additional peak in the PL spectrum (Figure 6e). The model thus suggests that the apparent splitting in PL spectra is not due to polaritonic states in the SC regime but is in fact due to the emergence of emission from the dark state as it gets coupled to the plasmonic cavity.

This model can explain also the observation of long lifetime measured in *g*^2^(*t*). The simulations show that the system possesses two decay channels (Figure 6f), but the femtosecond decay channel originating from the bright exciton coupling to the PC cannot be observed in the experiment due to experimental limitations, and only the slow component, attributed to the dark state, appears in correlation functions. The coupling of the dark state to the cavity shortens its lifetime through the Purcell effect.

In summary, we find that the involvement of additional excited states of the QDs leads to rich and complex excited-state dynamics, as bright and dark states couple differently to the cavity and contribute differentially to PL spectra.^3^

## 4 Conclusions and Prospects

In this Account, we described our studies on strong coupling between individual silver bowties and one to a few QDs. Scattering spectra of the coupled system demonstrated VRS, a manifestation of SC, even in the limit of an individual QE. EEL spectra of both bright and dark dipolar modes manifested splitting as well, providing strong evidence for SC. PL measurements on the same coupled system revealed several surprising observations, which were explained on the basis of the contribution of dark states of the QDs to the spectra. The coupling of a small number of QEs to plasmonic cavities thus exposes unexpectedly rich dynamics and emerges as an unconventional yet attractive means to control the dynamics of quantum states and potentially also chemical reactivity.

An important point to note is that in our studies with a single to a few QDs, the devices are situated close to the SC regime, though they often do not pass the somewhat arbitrary border between intermediate and strong coupling. An improvement of the preparative process of QD-PC devices should increase the coupling and facilitate reaching the SC regime. We demonstrated above one example for such an improvement, i.e., a reduction of the thickness of the adhesion layer deposited under the PC.^4^ An additional example of an improved preparative process involves using single-crystalline silver and ion milling to prepare the bowties.^51^ Grain boundaries and surface roughness in polycrystalline films are known to cause undesired scattering. Hence, using a single crystal of silver might reduce the plasmon line width. A different approach to increase the coupling is to hybridize a dielectric cavity with the PC.^52^ Hybrid dielectric-plasmonic cavities have demonstrated larger figures of merit *Q*/*V* and enhanced light matter interaction. Indeed, Gurlek at al.^53^ and Bisht et al.^54^ showed that embedding a PC in a Fabry–Pérot cavity facilitates entering the SC regime.

The ability to enhance further the coupling to single QEs might provide a route to affect their reactivity and manipulate their excited states.^21^ Indeed, PCs have been shown to suppress the photo-oxidation of organic chromophores.^55^ QE-PC coupling can also allow construction of efficient single-photon sources operating at room temperature, though whether these photons can reach a high-enough level of indistinguishability necessary for quantum information applications remains to be seen. Coupling to a PC might yield high rates of emission, and hence a high flux of individual photons.

The ability to strongly couple a few emitters to a PC paves the way to the observation of novel quantum optical phenomena. For example, when two or a few distinguishable QDs are positioned within the hotspot of a bowtie, and each QD is strongly coupled to the PC, the cavity may then mediate ultrafast interactions between them. Further, energy transfer between spatially separated QEs might be feasible when coupling them to delocalized optical modes such as surface plasmon polaritons or waveguide modes.^56,57^ To enhance the coupling in such devices, it would be interesting to consider hybridizing delocalized modes with intense localized modes within a PC. Such a hybrid plasmonic device was experimentally realized by Aeschlimann et al., who demonstrated SC of two whispering-gallery-mode antennas placed in the foci of an elliptical plasmonic cavity.^58^

Whether such and similar devices can contribute to applications in quantum information and quantum computing remains to be seen. However, as we hope to have shown in this Account, they already contribute to exciting science that involves bona fide nanoscale (i.e., deep subwavelength) energy exchange between QEs and cavities.
